# Guidelines for Optimal Patient Outcomes Using Calcium Hydroxylapatite for Jawline Contour

**DOI:** 10.1093/asjof/ojad019

**Published:** 2023-03-02

**Authors:** Amir Moradi, Jeremy B Green, Gideon P Kwok, Kim Nichols, Alexander Rivkin

## Abstract

**Background:**

Calcium hydroxylapatite (CaHA(+); Radiesse(+) [Merz North America, Inc., Raleigh, NC]) is the first FDA-approved injectable filler for subdermal and/or supraperiosteal injection to improve moderate-to-severe loss of jawline contour. CaHA has been recognized in the past for its ability to provide contour and support overlying tissues and utilized for jawline augmentation well before this recent indication; however, with recent FDA approval of CaHA(+) for jawline contour improvement, it is important that clinicians are aware of best practices for patient selection, treatment planning and injection, as well as safety considerations and postprocedure care.

**Objectives:**

To provide guidance on best practices for patient assessment and on-label use of CaHA(+) for jawline rejuvenation and augmentation.

**Methods:**

As part of a 2-h roundtable discussion, 5 clinicians with expertise in both the use of CaHA(+) and jawline treatment discussed patient selection, CaHA(+) injection technique, and important safety measures, with the purpose of developing guidance to support optimal clinical use.

**Results:**

The most common applications of CaHA(+) in the jawline are rejuvenation of the prejowl sulcus, recontouring the jawline, and providing definition to the gonial angle. Improving the gonial angle is of particular interest as it is a procedure sought by patients of all genders and ages. Variations in technique are discussed and case studies are presented.

**Conclusions:**

Jawline augmentation is a procedure with wide-ranging appeal for a diverse array of patients. CaHA(+) is an ideal filler for jawline augmentation due to its rheologic properties (high *G*′) and ability to achieve defined contours and angles. Appropriate injection technique permits effective treatment and outcomes associated with high patient satisfaction.

**Level of Evidence: 5:**

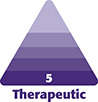

The development and adoption of minimally invasive modalities for improving facial contour and volume has led to an increased interest in aesthetic procedures, and in 2020 the number of noninvasive procedures outpaced surgical procedures (13,281,235 vs 2,314,720, respectively).^1^ Dermal fillers are second only to botulinum toxin as the most popular noninvasive aesthetic treatments, with hyaluronic acid (HA) and calcium hydroxyapatite (CaHA and CaHA(+); Radiesse and Radiesse(+) [Merz North America, Inc., Raleigh, NC]), ranking as the most popular fillers.^1,2^

CaHA/CaHA(+) is a biodegradable filler that contains 25-45 µm CaHA microspheres composed of phosphate and calcium ions (the main minerals in teeth and bones)^3^ suspended in a carboxymethylcellulose-based carrier gel.^4–6^ The CaHA(+) formulation differs from CaHA in that it also contains 0.3% integral lidocaine hydrochloride for improved patient comfort; however, the formulations have similar clinical performance and flow properties.^4,5,7^ CaHA and CaHA(+) are unique among biostimulatory fillers in that they provide volume through a dual mechanism which includes both the provision of immediate volume through the lifting capacity of the CaHA microspheres and carboxymethylcellulose gel, as well as regeneration of multiple components of the extracellular matrix.^4–6^ As the carrier gel dissipates, the microspheres interact with the tissue matrix, directly stimulating dermal fibroblasts to produce endogenous collagen types I and III, increasing elastogenesis, and additionally increasing proteoglycans and angiogenesis.^8–10^ Togehter, these activities not only give rise to a longer-term aesthetic correction, but also improve the quality of overlying skin.^6,8,10,11^ Importantly, the high *G*′ and viscosity of CaHA and CaHA(+) make this agent well-suited for placement on periosteum, where it can lift overlying tissue and serve as a modulable implant that retains shape, which uniquely allows for a defined edge and projection. Clinically, CaHA/CaHA(+) is an ideal product for replacing volume and bony support that have been lost, primarily due to its capacity to provide structural support. Thus, CaHA(+) is and is particularly well-suited for the augmentation of jawline contour.^8,12–14^

The jawline is an area of the face especially prone to exhibiting signs of aging as a result of skeletal changes and soft tissue volume loss. Overall, facial aging is a multifactorial process that is caused by a combination of elastin, collagen, and glycosaminoglycan breakdown; fat displacement and decreases in volume; changes in muscle tension and length; and resorption of bony structures.^15–17^ One of the most notable changes in facial structure that occurs with aging is a loss, lack, or blunting of the mandibular angle as well as loss of definition along the jawline, which occurs due to a combination of skeletal remodeling and resorption as well as tissue laxity and loss of volume in the fat pads of the lower face.^16^ With age, the chin also shortens, resulting in decreased projection, which can affect the apparent length of the mandible.^16^

The contribution of jawline contour to facial attractiveness is substantial, and a wide range of patients (all genders and across a large age range) frequently request treatments aimed at jawline beautification/aesthetic improvement and/or rejuvenation. Patients frequently request a taut jawline (also known as a “snatched” jawline) that clearly delineates the face from the neck, and gives rise to a shadow cast from the jawline onto the neck. For female patients of any age, an elegant, well-defined jawline is a beauty standard prized all over the world, while for male patients, a prominent, broad and straight jawline is considered a strong indicator of masculinity and has been correlated with overall health and traits of competence and leadership.^18,19^ Interest in jawline injection among males is increasing, and this treatment represents a unique opportunity as an entry procedure to aesthetics for these patients.

Recognizing both the increasing interest in jawline contour and the recent approval of CaHA(+) for this purpose, the authors participated in a roundtable on best practices for the treatment of the jawline with CaHA(+) to share their approaches to optimizing patient outcomes and satisfaction. These guidelines are intended to aid aesthetic clinicians in the on-label use of CaHA(+) for improving jawline contour. Included herein is practical guidance for appropriate candidate assessment and selection, injection technique, and a detailed discussion of the necessary safety precautions to be taken to avoid complications.

## METHODS

**Figure 1.**
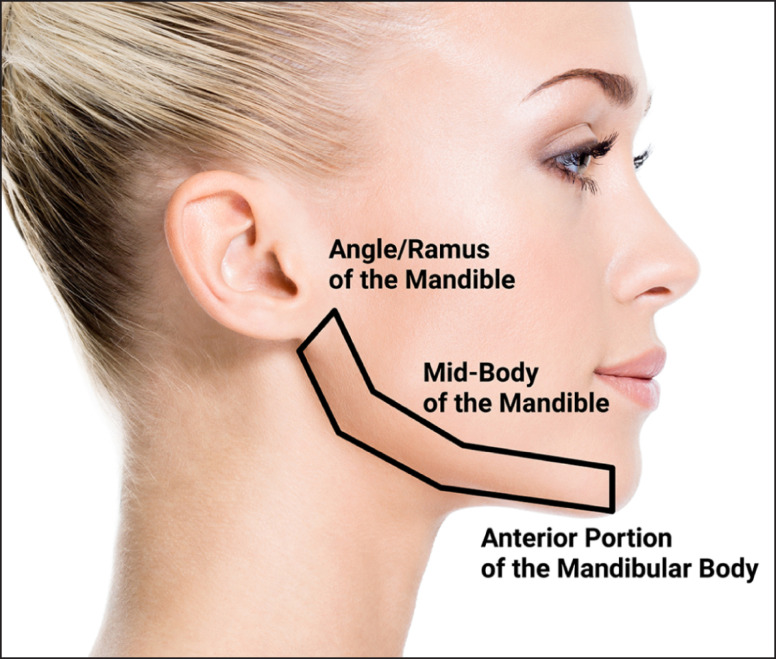
On-label treatment areas for jawline augmentation with CaHA(+): the angle of the ramus of the mandible and the mid-body of the mandible to the anterior portion of the mandibular body are indicated. Created by and published with permission from James Silvera.

As part of a roundtable held in May 2022, a team of 5 experts in aesthetic medicine convened to discuss the on-label use of CaHA(+) to improve moderate-to-severe loss of jawline contour (Figure 1). The discussion included an overview of patient selection, education, expectation setting, treatment planning and injection, as well as safety considerations and postprocedure care. The authors also shared instructional videos that detail recommended injection techniques and guidance on safety. All patients treated as a part of this activity provided written informed consent and were treated in agreement with the principles outlined in the Declaration of Helsinki. All patients whose images are shown here signed releases granting permission for their photographs and/or videos to be displayed.

## RESULTS

### Patient Selection

**Figure 2.**
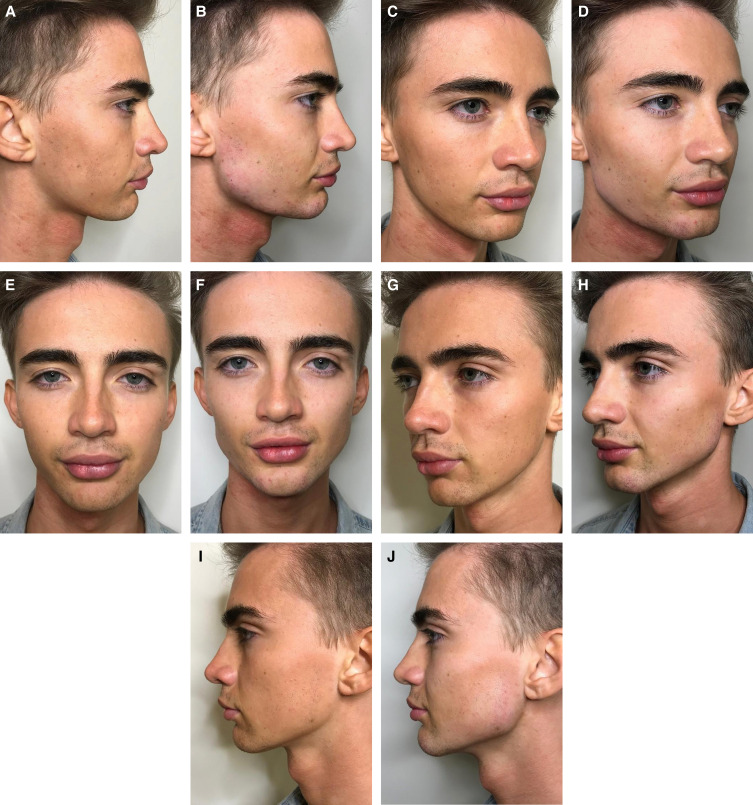
A 25-year-old male at (A, C, E, G, I) baseline and (B, D, F, H, J) immediately after treatment with 7.5 cc of CaHa(+) in the jawline.

While all patients generally desire a taut jawline, patients who typically seek jawline rejuvenation/remodeling with dermal fillers can be considered as two distinct groups. More mature patients (>40 years of age) are most likely to present desiring correction of jowling and/or loss of definition along the jawline, while younger patients (22-40 years of age) are more likely to present desiring sculpting to correct a lack of contour or to improve facial symmetry. Importantly, jawline sculpting and contouring is a somewhat unique procedure in that it is actively sought by patients desiring a jawline with masculine geometry and can draw patients to the clinic for treatment who might not otherwise seek aesthetic care thereby serving as an introduction to aesthetic treatments in general.^20–22^ In Video 1, the ability of CaHA(+) to provide a distinct edge and structure to a poorly defined jawline in a younger male patient is shown. This patient is also shown in Figure 2.

The ability to shape the face is also important for female-to-male transgender and gender-fluid individuals.^23,24^ Given the issues many transgender and gender fluid patients face with insurance coverage for surgical procedures,^25^ jawline rejuvenation with dermal fillers may provide a more accessible, nonsurgical alternative to create a jawline that aligns with their gender and desired appearance.

Regardless of population, certain patient features make jawline enhancement more or less likely to be successful (Table 1). Treatment success and patient satisfaction are likely to be highest in patients with good skin quality and collagen-producing capacity without excessive skin laxity. An ideal mature candidate has some visible, but not excessive, jowling and a moderate, but not severe, loss of jawline definition. More mature patients with overt jowling may also benefit from filling of the pre and postjowl sulci and enhancement of the angle of the mandible; however, expectations should be realistic. Irrespective of age, patients with significant skin laxity or excess fatty tissue in the neck are unlikely to achieve optimal results with filler injection alone. A combination treatment plan may be more appropriate for some patients, with skin tightening treatment(s) as a first step for managing laxity or, in cases of excess adipose tissue in the neck, management of excess tissue (eg, liposuction, etc.), before jawline augmentation. For patients with round faces, dermal fillers should be used only in select cases in which adding volume to the angle of the mandible results in a more angulated and sculpted appearance.

**Table 1.** Features of the Ideal Patient Indicators/Contraindications for Jawline CaHA(+) Administration

Patient type	Features
Ideal mature patient	Patients seeking to camouflage minor jowling caused by volume loss
	Patients seeking to replenish lost volume along the length of the jawline
	Patients seeking subtle lift of the jawline
	Patients seeking to straighten or better define the jawline
Ideal younger patient	Patients seeking sculpting/definition of the jawline
	Patients seeking to optimize jawline proportions/symmetry
Nonideal patient	Patients with significant jowling or heavy skin
	Patients with excess skin laxity in the lower face
	Patients with excess adipose in the neck
	Patients with a square jaw for whom widening the face would be detrimental

CaHA(+), calcium hydroxylapatite.

### Treatment Planning

Before injecting, it is important to discuss treatment goals with the patient. Optimal correction of the jawline can require a greater volume of product than other areas of the face, so it is particularly important to ensure the patient understands the cost of the desired outcome. Although it is important to consider what a patient may be able to afford when creating a treatment plan, it is critical that the clinician resist pressure to deliver less treatment than is optimal in the jawline area due to cost. It is better to have the patient come back when they can afford a full procedure than do less than enough and have the patient suffer inappreciable results. Of note, a top-down assessment of the face should be performed, as indirect effects from managing the midface may alleviate some inferior descent of the tissues in the lower face, thereby decreasing the filler needed to camouflage the jowls. Further, management of these areas may help maintain ideal facial proportions and harmony, facilitating a balanced, global improvement.

The following sequence provides optimal and safe delivery of CaHA(+) for treating the jawline. A discussion of treatment planning and relevant anatomy is included in Video 2:

#### General Guidance

- Standardized before and after images should be captured to track treatment-related changes. Ideally, 5 angles should be captured: front and both profile and oblique views.
- A needle and/or cannula can be used to inject the jawline depending on the comfort level of the injector. Irrespective of technique, an awareness of relevant anatomy and vasculature is requisite.
- While the sections below detail the technique for needle and cannula within distinct planes, it is important to note that multiple injection depths may result in better tissue projection and more significant augmentation.
- It is important to avoid volumizing the jowl fat compartment lateral to the mandibular ligament, as this will create heaviness: this area may be considered an “aesthetic danger zone”.
- In patients with jowling, treating the mandibular angle in the postjowl area, not just in the prejowl sulcus is important for fully camouflaging descended fat pads.

### Injecting the Angle of Mandible

**Figure 3.**
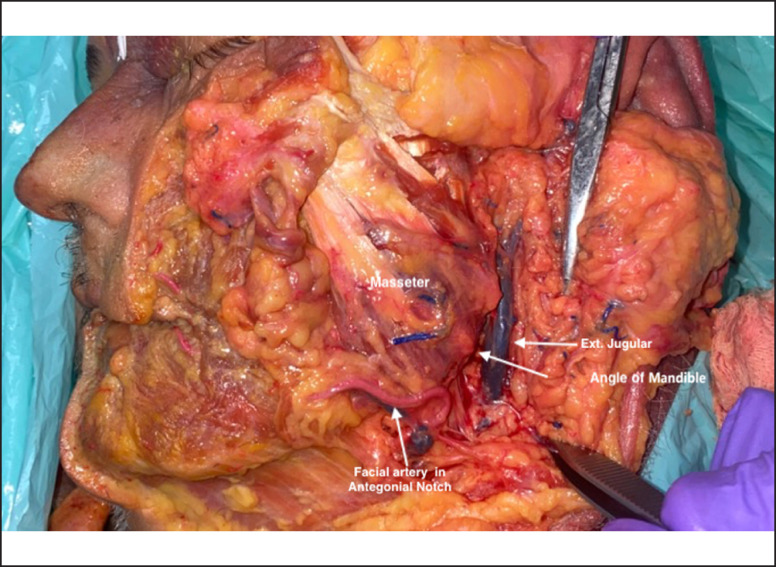
Cadaver dissection showing the relevant anatomy of the mandible.

**Figure 4.**
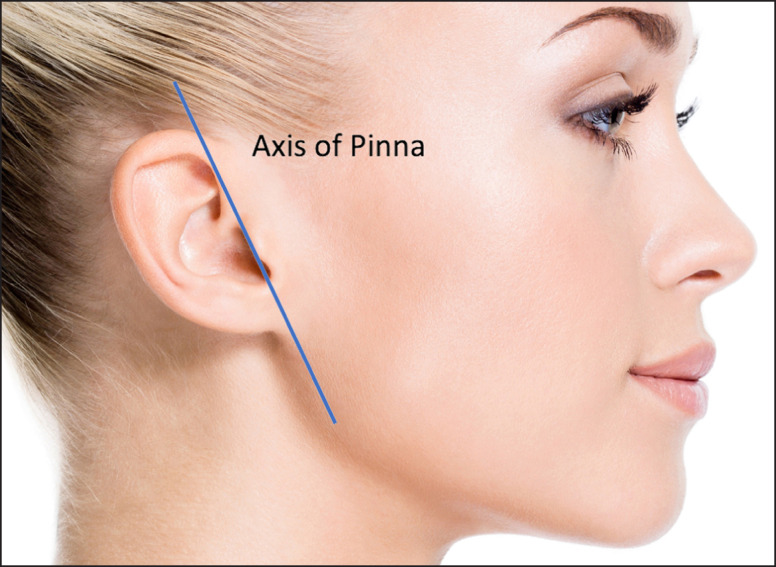
The axis of the ear (Pinna) can be used to determine the ideal position of the mandibular angle posteriorly. Created by and published with permission from James Silvera.

- Before injecting, palpate the antegonial notch (Figure 3). Locating this structure is important for ensuring that the facial artery is avoided during deeper injection.^26^
- Manually palpate and isolate the angle of mandible (AOM). One may place a finger on the ramus and a finger on the body to feel for the angle. In most cases, the angle of mandible is managed first, followed by sculpting the remainder of the jawline.
- Use the axis of the ear (Pinna; Figure 4) to determine the ideal position of the mandibular angle in the antero-posterior plane.
  - The feminine AOM should most often lie just anterior to where the axis of the ear intersects with the line of the mandibular body. To emphasize the more feminine aspect of the jawline, the axis of the ear should be the posterior limit of jawline augmentation. Extending the injection beyond this line results in a narrower AOM, which can create a broader and more square jaw. A jawline with a wider AOM can have a softer, more feminine appearance, and to acheve this effect, CaHA(+) is placed a bit more superior and medial (less projected).
  - In a squared, or more masculine jaw, the AOM can lie on the intersection between the axis of the ear and mandibular body, thereby forming a slightly less obtuse angle. For a squarer, broader jawline, the clinician may need to extend the angle of the mandible to the axis of the ear and place the injections slightly posterior.

The AOM can be injected with either a cannula or a needle. While in clinical studies, patients were randomized equally to be injected with a cannula or needle, injection with a needle is more common in clinical practice. When injecting with a needle, injections should be placed deep as columns in a retrograde fashion, starting from the supraperiosteal plane and stopping at a deep point in the subcutaneous layer. Placement of a thick product like CaHA(+) in deeper planes gives the injector the best chance of an excellent outcome and smooth final contour because there is more tissue overlying the filler. Putting excessive volumes of filler in the superficial layers risks a result with an irregular contour and unnatural appearance. Deep injections are also safer at all locations along the mandible, except the antegonial notch, where the facial artery crosses the mandible and travels just over the bone (Figure 3). The volume of each injection should be approximately 0.2 cc or less and the needle should always be moving in order to mitigate risk of vascular events. Injections should also be performed slowly with the least amount of plunger pressure possible.

The jawline and AOM can also be injected using a cannula. As outlined above, it is easier to get a smooth outcome with deep plane placement, so this should be the plane for beginner to intermediate cannula users. Subcutaneous injection can also be part of an effective approach, but care must be taken to spread the product, so it blends flawlessly with the surrounding tissues. Jawline augmentation with a cannula in the superficial plane should only be performed by expert cannula injectors. The first entry point for the cannula should permit injection of the AOM as well as a portion of the jawline. The cannula is advanced from the point of entry, using the nondominant hand to pull the overlying skin taut can facilitate its passage, and CaHA(+) is deposited in a thin layer using a retrograde fanning technique and massage following placement to ensure a smooth surface contour. Importantly, in the subcutaneous layer the septa dividing the fat compartments can create some resistance, and passage through these structures is needed in order to treat the surface of the entire jawline.

### Injecting the Pre and Postjowl Areas

Depositing the product at the post- and prejowl areas can help camouflage the jowl and give the jawline a straighter appearance. Beginning at the AOM, just ahead of the posterior edge, injections should follow along the periosteum until anterior to the prejowl sulcus, taking care to avoid the facial artery. Using a needle, CaHA(+) is placed along the jawline using a retrograde threading technique. The needle is inserted at a 45° angle to the skin. Once it reaches periostum, the needle angle is brought down to about 10° and the needle is advanced along the bone. A smooth strip of CaHA(+) is placed with minimal plunger pressure along the bone as the needle is withdrawn slowly. If the pre- or postjowl sulcus is particularly deep, columns of filler can be placed in a fashion similar to that described for the AOM above with the columns of filler oriented in the direction of desired tissue projection (inferior, lateral, or both).

If using a cannula, the product may be deposited using a tunneling and linear threading or retrograde fanning technique in the deep or subcutaneous plane, as described for the AOM, above. As with the AOM, deep placement and abundant overlying tissue make a smooth outcome easier to achieve. The filler may be placed along the jawline, but fanning may be done more superiorly within the regions outlined in Figure 1, in order to create vectors and to ensure an even, blended contour. Along the mandible, the subcutaneous plane permits avoidance of major vessels (ie, facial artery and vein), which are deeper, beneath the platysma.

**Figure 5.**
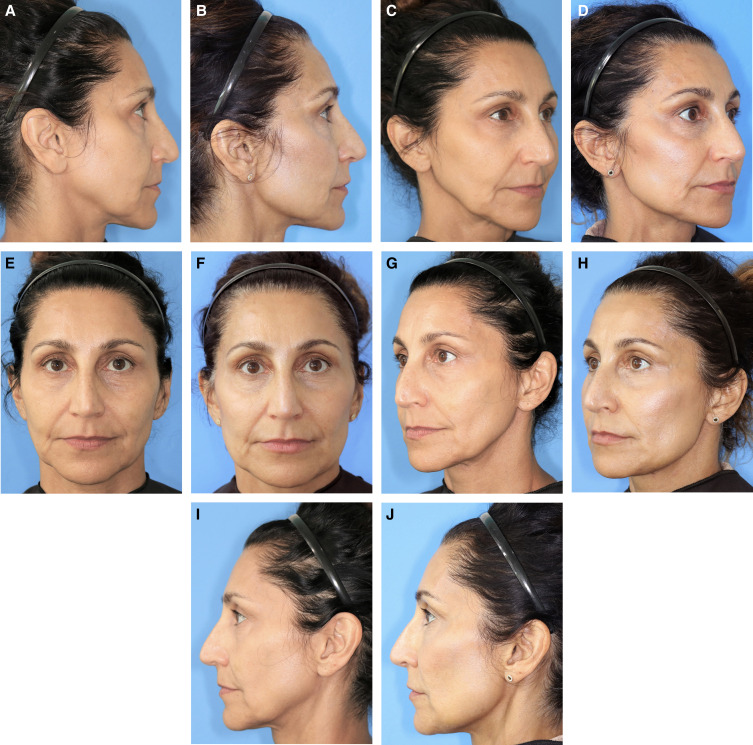
A 52-year-old female at (A, C, E, G, I) baseline and (B, D, F, H, J) 2 weeks after treatment with 4.5 cc of CaHA(+) (2.25 per side) in the jawline.

**Figure 6.**
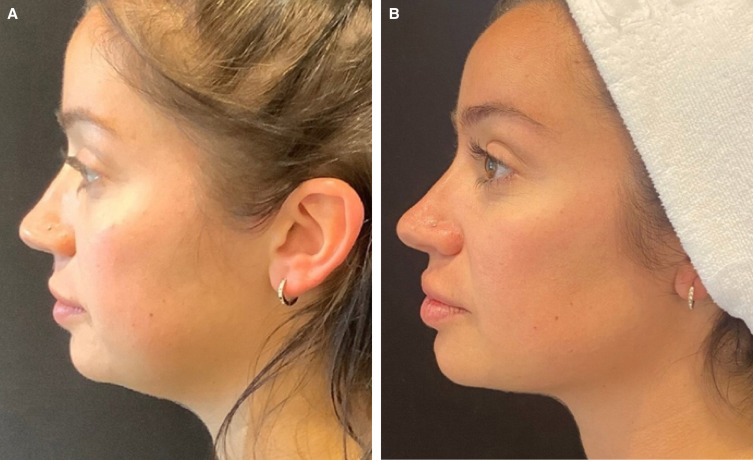
A 30-year-old female at (A) baseline and (B) 2 weeks after treatment with 1 syringe (1.5 cc) per side of CaHA(+) in the jawline only. A lift in submental tissue posttreatment is clearly observed.

**Figure 7.**
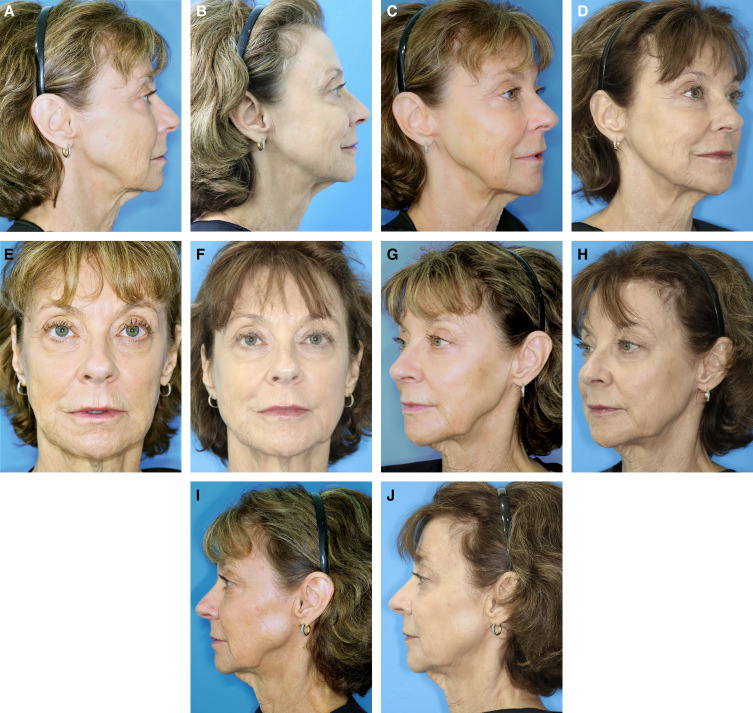
A 64-year-old female at (A, C, E, G, I) baseline and (B, D, F, H, J) 2 weeks after treatment with 3.0 cc of CaHA(+) per side in the jawline. For enhancement of a softer jawline, approach, but do not pass posterior to, the axis of the ear to avoid a square jawline in the lateral profile.

As with any injection, jawline treatment must be modified to suit patient needs. Video 1 shows patient evaluation and injection technique for improvement of the gonial angle in a male patient; Video 2 (patient also shown in Figure 5), shows injection techniques for jawline enhancement of a female patient with a moderate need for improvement of jawline contour; and Videos 3 and 4 both show treatment planning and injection of the prejowl sulcus in a more mature patient. Figures 6 and 7 show outcomes following treatment with CaHA(+) in the jawline. For the patient shown in Figure 7, a video of the injection is shown in Video 5.

### Safety and Postprocedure Care

CaHA(+) jawline treatments have a favorable safety profile. Most adverse events are minor, transient, and resolve without intervention.^8,27,28^ However, practitioners should be aware of the vasculature and anatomic structures present in this area, as well as how to deliver the product safely. Care must be taken to avoid the facial artery, which arises from the external carotid artery just distal to the lingual artery in the carotid triangle and ascends obliquely beneath the digastric and stylohyoid muscles and crosses over the mandibular body at the antegonial notch, just anterior to the masseter muscle along the surface of the mandible (Figure 3). Injections within this area should be subcutaneous, permitting avoidance of major vessels.

In addition to avoidance of vasculature, slow injection of small volumes and continuous motion while injecting are important procedural points which improve safety. The injection of smaller volumes not only prevents the deposition of large volumes within a vessel should it be inadvertently encountered, but also prevents focal accumulation of product, which can occur with large volumes of CaHA and CaHA(+) due to its high viscosity.^29^

As discussed above, the authors vary with regards to preference for using a cannula, needle, or both to inject CaHA(+). One author noted that he often uses a cannula to deliver CaHA(+) within the prejowl sulcus at the mandibular ligament (ie, the attachment between the undersurface of the dermis and the bone), and a needle for more precise placement filler at the inferior edge of the mandible and for contouring at this location. Though both cannulas and needles are on-label administration methods, each has a distinct set of advantages, summarized in Table 2. Importantly, while 25-gauge needles and cannulas allow for controlled product deposition, a 22-gauge cannula is preferred because it is far less likely to enter a blood vessel.^30^ Care must be taken with 22G cannulas, however, as it is easier to extrude a large volume of product quickly. This can be disastrous if the cannula somehow pierces a vessel (extremely unlikely if other safety protocols such as slow, soft injection with minimal pressure are followed) and can also result in a bumpy, irregular final contour.

**Table 2.** Benefits of Needles vs Cannulas

Benefits
Needles
More effective for entering a specific tissue location/depth More precise Allow the injection of small volumes of product Relatively higher risk of penetrating a vessel
Cannulas
Allow the diffuse distribution of product over a wider surface area Less precise product placement Associated with less pain, swelling, and bruising due to fewer injection sites More effective for threading, fanning, or “pearling” product Less likely to penetrate a vessel

The majority of adverse events associated with CaHA(+) are short-term (ie, <7 days), injection site-related reactions (ie, ecchymosis, edema, erythema).^4,5^ In clinical studies of CaHA(+) for jawline augmentation, only 17.2% of adverse events (including but not limited to pain, lumps/bumps, redness, and itching) persisted past 2 weeks. For the only other FDA-approved product for jawline (ie, vycross HA, VYC-25), 35% of reported injection site reactions lasted over 2 weeks through 1 month.^31^

In the experience of the authors, the most common side effect about which it is important to educate patients is injection-associated swelling, which takes approximately three days to dissipate. Bruising is a risk with all soft tissue fillers, but for jawline augmentation, bruising is more likely to be associated with fanning and threading than with deep injections into the preperiosteal plane.^32^ Nodules or lumps are uncommon and are most often the result of overcorrection, overly superficial filler placement, or failure to evenly distribute product while injecting.^33^ Though vascular compromise is uncommon, it is a serious complication: for this type of adverse event, prevention is critical and a knowledge of anatomy and recommended treatment protocols is requisite.^29,34^ Open-access publications are available that recommend treatment algorithms for a variety of adverse events as a result of filler treatment^33,35,36^ as well as protocols specific to CaHA.

## DISCUSSION

A well-defined and balanced jawline is a hallmark of beauty and can define perceptions of masculinity, femininity, and youth. Further, adequate management of the lower face is critical for achieving a balanced and harmonious appearance. The rheologic properties of CaHA(+), in particular its high *G*′ and lifting capacity make it uniquely suitable for providing the lift, projection, definition, and contouring needed for augmentation of the jawline. While HA filler may provide volume enhancement to the jawline, the low *G*′ results in a more rounded border. In contrast, CaHA(+) may provide an edge that results in shadowing beneath the jawline onto the neck and gives patients a so-called “snatched” jawline.

Jawline augmentation and/or rejuvenation have a wide-reaching appeal. For some patients, jawline treatment represents an entry point for them into the aesthetic marketplace and an opportunity to establish a relationship with patients who may be interested in preventive or more proactive management of aging. Improvement of the gonial angle (angle of mandible) and establishment of a more robust and defined jawline is commonly requested by male patients and represents an important opportunity to treat this patient population. Younger patients who do not yet need other aesthetic treatments are opting for jawline sculpting as well. Importantly, transgender individuals who do not have access to transition surgery may find contouring of the jawline with CaHA(+) to be an effective and accessible way to address their transgender-associated body dysphoria.^23–25,37^

While treatment protocols and primary principles of treatment are outlined here, tailoring the injection pattern to the individual needs of the patient is critical. Despite variations in the authors’ techniques, safety is always a critical consideration and constant focus. A thorough understanding of facial anatomy is requisite, and all procedural precautions should be taken (administration of small aliquots of product, slow injection with continuous movement). In the experience of the authors, aesthetic results with CaHA(+) for jawline treatment are excellent, and patients are delighted with the procedure.

## CONCLUSIONS

CaHA(+) is a high *G*′, regenerative filler that permits sculpting and correction of contouring of the jawline as well as provision of lift and definition. Depending on administration, this treatment may also support the feminization or masculinization of the jawline based on an individual patient's needs. This report builds on the extensive experience of experts in jawline treatment with CaHA(+) to provide guidance for on-label use.

## Supplementary Material

ojad019_Supplementary_Data
